# Sustainable Valorization of Plastic Waste and Palm Fronds into Chemically Activated Carbon–Polymer Composite

**DOI:** 10.3390/polym17172356

**Published:** 2025-08-29

**Authors:** Junaid Saleem, Zubair Khalid Baig Moghal, Furqan Tahir, Gordon McKay

**Affiliations:** 1 Division of Sustainable Development, College of Science and Engineering, Hamad Bin Khalifa University, Qatar Foundation, Doha P.O. Box 5825, Qatargmckay@hbku.edu.qa (G.M.); 2 Center for Advanced Materials, Qatar University, Doha P.O. Box 2713, Qatar

**Keywords:** plastic waste, valorization, palm fronds, activated carbon, environmental impacts, energy net, life cycle assessment, climate change

## Abstract

Polyolefin waste is an abundant yet underutilized resource for developing value-added materials, while palm fronds (PF), a lignocellulosic biomass, offer a promising feedstock for activated carbon (AC) production. However, conventional AC from biomass is typically obtained in powdered form, making it difficult to handle and recover in aqueous systems without external support. Incorporating polyolefins during synthesis enables the formation of chemically activated polymer–carbon composite (PCC), which offers improved usability and recovery. This study aims to evaluate the environmental sustainability of producing PCC from PF and polyolefins, using Life Cycle Assessment (LCA) to quantify energy consumption and climate change impact. The LCA results show a net energy demand of 88.59 MJ and a climate change impact of 3.57 kg CO_2_ eq. per kg of PCC. Substituting conventional petroleum-based AC with PCC led to a 28% reduction in climate change impact and a 30% decrease in energy demand. By integrating biomass and plastic waste, this research supports sustainable material development and promotes circular economy practices in water treatment applications.

## 1. Introduction

Polyolefins, including polypropylene (PP) and polyethylene (PE), make up the largest proportion of mixed plastic waste (MPW) [1,2]. In 2022, the global production of PE and PP reached approximately 110 MMT and 79 MMT, respectively, with projections indicating growth to around 135 MMT and 105 MMT by 2030 [3,4]. Despite their widespread use, less than 10% of these plastics are recycled [5,6]. A report from 2017 highlighted that plastic packaging materials lose 95% of their value after a single use, resulting in an annual economic loss of USD 120 billion [7]. These figures reflect not only the growing environmental burden but also the pressing need to develop sustainable strategies for valorizing polyolefin waste.

One promising strategy for addressing both plastic and agricultural waste challenges is the integration of polyolefin plastics into the production of activated carbon (AC), a highly porous and reactive material widely used in water and air purification [8,9]. Biomass-derived AC presents a renewable, low-cost alternative to conventional petroleum-based AC and aligns with circular economy goals by utilizing agricultural residues [10,11] including date pits [12,13,14,15], palm fiber [16,17], pomace leaves [18,19,20], date palm fronds (PF) [21,22], olive stones [23,24,25], bamboo [26], Azolla filiculoides [27], and coconut shells [28,29]. PF, in particular, is an abundant lignocellulosic waste with proven potential as a feedstock for AC, yielding a product with excellent adsorption properties following pyrolysis and chemical activation. However, biomass-based AC is typically obtained in fine powdered form, which complicates its recovery from aqueous environments. To overcome this, incorporating thermoplastic polyolefins during synthesis enables the formation of flake-like AC structures that are easier to handle and separate in water treatment processes. This co-processing approach not only enhances the practicality and reusability of AC but also offers a novel route to valorize low-recyclability plastic waste, thereby addressing the environmental limitations associated with both AC production and plastic disposal.

To address the handling and recovery limitations of powdered activated carbon (AC), recent studies have proposed structural modifications such as granular AC [30], composites and membranes [31,32], carbon monoliths [33,34,35], and flake-like structures [36]. While these approaches improve usability, they often involve complex fabrication techniques or incur high costs. A simpler and more scalable alternative is the incorporation of polyolefins—such as PE and PP—into the AC synthesis process. Their thermoplastic and adhesive properties facilitate the formation of structured flakes from powdered AC, improving separation and reusability in aqueous systems. This co-processing strategy not only enhances the functional performance of biomass-derived AC but also contributes to the recycling of polyolefin waste, which is typically difficult to manage due to its chemical inertness and low recycling rates [37,38]. By combining biomass and plastic waste, this approach supports circular economy principles and advances practical solutions for water treatment.

Despite these advantages, limited research has focused on the integration of palm fronds (PF) and polyolefins into a unified synthesis route for producing structured activated carbon (AC) materials. Additionally, most existing studies emphasize adsorption performance, while few address the environmental implications of the synthesis routes. A rigorous environmental performance assessment using Life Cycle Assessment (LCA) is therefore the core objective of this work.

This study aims to evaluate the environmental sustainability of producing a chemically activated polymer–carbon composite (PCC) from PF and polyolefins, using LCA to quantify energy use and climate change impact. Specifically, it develops a four-step open-loop recycling method combining PF and polyolefins to produce PCC. The results show that PCC offers superior environmental performance compared to petroleum-derived AC. Furthermore, the effect of different energy sources—including natural gas, solar photovoltaics, solid biomass, hard coal, and hydropower—was analyzed to identify opportunities for further sustainability improvements.

## 2. Materials and Methods

Potassium hydroxide (KOH) and sodium hydroxide (NaOH) were purchased from Sigma Aldrich, St. Louis, Missouri, MO, USA. PF were collected locally. Desalinated and deionized water was used throughout the experiment, including activation and neutralization steps. An isomeric mixture of xylene was purchased from VWR Chemicals, UK, and used as received. Waste milk bottles (high-density polyethylene, HDPE) and food containers (polypropylene, PP) were combined as polyolefin waste, while ultrahigh molecular weight polyethylene (UHMWPE) was obtained from Sigma Aldrich.

XRD patterns were obtained using a Malvern Panalytical EMPYREAN diffractometer by Malvern Panalytical, Malvern, UK, operating with Cu Kα radiation (λ = 1.5406 Å) at a scanning rate of 2°/min over a 2θ range of 5° to 80°.

FTIR spectra were recorded using a PerkinElmer Frontier FTIR spectrometer by PerkinElmer (Hopkinton, MA, USA) in the range of 4000–500 cm^−1^, using the ATR (attenuated total reflectance) mode at a resolution of 4 cm^−1^. Scanning electron microscope (SEM) images were captured with FEI Quanta650FEG by Thermo Fisher Scientific (Waltham, MA, USA) after sputtering with gold layer of 5 nm.

### Preparation of PF Activated Carbon-Polymer Composite 

**Figure 1:**
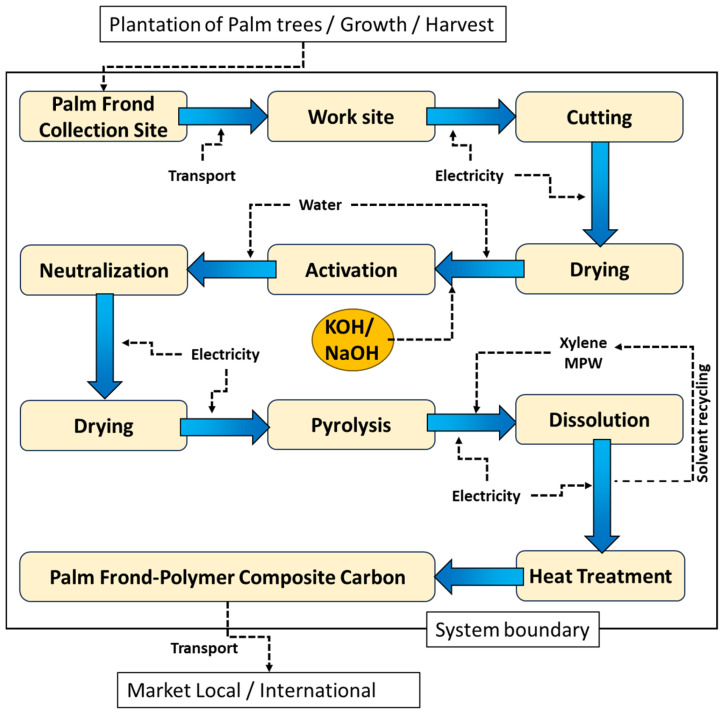
The process flow diagram of the preparation of PCC.

The process flow diagram for the preparation of PF Activated Carbon-polymer composite is shown in Figure 1. The process begins with the collection of PF, which is transported to the workplace using a diesel-powered truck (single unit truck, USA). The PF is then shredded into small pieces (1–2 cm) and dried to remove residual moisture in hot air oven, this drying step can also be performed under sun to further reduce emissions, but consumes more time. The quantities of materials and the heat requirements for each step are detailed in Table 1.

The dried PF is activated using two different chemical routes: KOH and NaOH. For activation, 2.0 M solutions of KOH and NaOH were prepared using deionized water. The dried PF were dipped in basic solution for overnight and neutralized with water to remove excess or loosely attached base from the surface. Then, the activated PF were dried in hot air oven and subjected to pyrolysis. The dried PF were kept in the furnace for three hours at 600 °C with an increase in temperature from 30 to 600 °C at a rate of 20 °C per min. During pyrolysis, the activated PF is converted into AC.

To prepare the polymer–carbon composite, a flask containing a semi-crystalline polymer was taken and a solvent was added, and the flask was heated to dissolve the polymer. The amount of polymer and its composition is presented in Table 1. The temperature is maintained below the boiling point of the solvent, and the setup is kept in a closed container to prevent evaporation. Once the polymer dissolves, a measured amount of AC is added to the hot solution and stirred to ensure uniform dispersion. The solution is then poured into a mold of the desired shape and allowed to stir to disperse homogenously, then the reaction solution was cooled down to room temperature, during which the polymer–carbon composite solidifies, and the solvent separates. Any entrapped solvent is removed using a vacuum condenser. Finally, the composite was subjected to heat treatment (up to the melting point of the polymer) to enhance its strength (improves the binding of the composite), yielding the final PF-based PCC.

All data were collected from experiments, the literature, or the ‘LCA for Experts’ software v6.5 (Sphera) and database (MLC Databases Edition 2024). LCA, a standardized methodology for evaluating the environmental footprint of products and processes [39,40,41,42], was employed to analyze the EI of PCC production. LCA involves four steps: goal/scope definition, inventory compilation, impact assessment, and interpretation, following ISO 14040/14044 standards [43,44,45]. The EI data for commercial AC were sourced from the ‘LCA for Experts’ database. While multiple EI categories were examined, the study focused primarily on Energy Net (EN) and climate change (CC). Table 2 presents the selected EI, their corresponding units, and abbreviations used consistently throughout the study. The ReCiPe midpoint approach was utilized to assess the EI associated with PCC production. Also, the following assumptions are made.

The quantification of inputs and outputs is derived from lab-scale experiments, with all materials for the recycling process sourced locally. Deionized desalinated water is used in place of desalinated water or groundwater wherever necessary. Emissions and energy consumption associated with equipment usage are not included in the analysis. The functional unit is defined on a mass basis as 1 kg of PCC produced from PF and polyolefin waste.

## 3. Results and Discussion

**Figure 2:**
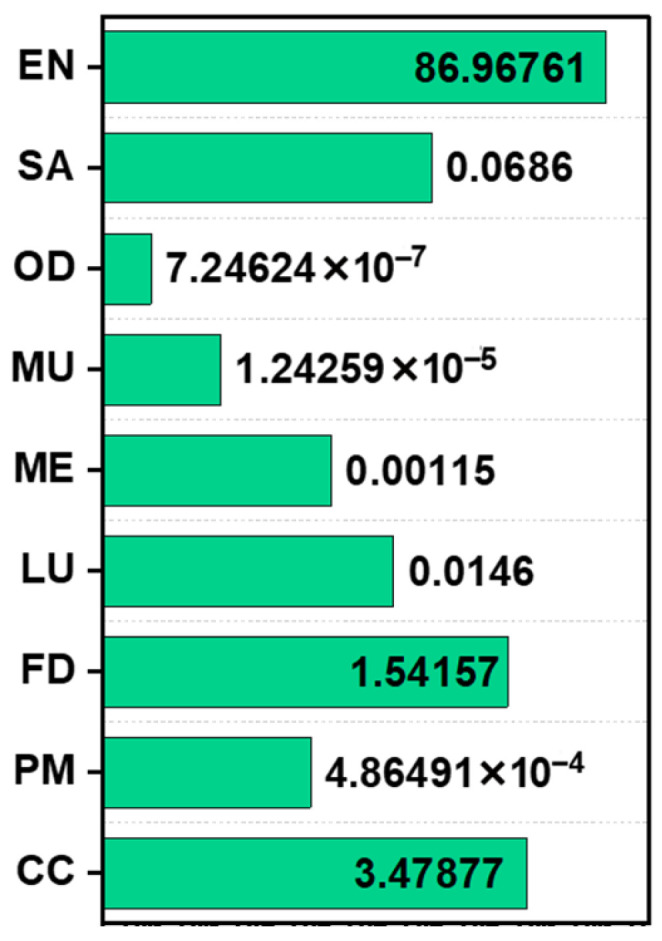
EI of PCC.

We first determine which activation pathway results in lower EI by comparing KOH and NaOH. After finalizing this choice, we proceed with the rest of the study using NaOH. The EI of KOH and NaOH were assessed using natural gas as the energy source and are presented in Table 3. The results indicate that KOH has a higher net energy demand (88.59) compared to NaOH (86.97) and a higher climate change impact (3.57) than NaOH (3.48). This difference is attributed to the more energy-intensive production process of KOH as it is manufactured through the electrolysis of potassium chloride (KCl), a process that demands significantly more energy than the membrane electrolysis of sodium chloride (NaCl) used for NaOH production. This increased energy requirement stems from KOH’s high reactivity, as it readily absorbs carbon dioxide and water, necessitating a more intensive operational process. Furthermore, the electrolysis of KCl generates KOH, chlorine, and hydrogen, consuming more electricity than NaOH production, which efficiently separates NaOH and hydrogen from chlorine with minimal risk of hazardous by-products. Additionally, the higher cost and lower natural abundance of KCl compared to NaCl further contribute to the increased energy consumption and carbon emissions associated with KOH production. LCA results for all import impact categories are reported, paying specific attention to EN and CC, as presented in Figure 2.

A key distinction between this work and our earlier preliminary study [17] is the choice of biomass feedstock: palm fronds (leaflets and petioles, cellulose/hemicellulose-rich and porous) versus palm fibers (dense husk material with higher lignin content), which directly affects carbon yield, activation efficiency, and life-cycle impacts. The fiber-based study [17] mainly served as a proof-of-concept, demonstrating flake formation with adsorption capacity of 200–240 mg/g but offering only a limited LCA scope. In contrast, the present palm frond–based study achieved lower energy demand (88.59 MJ vs. 103.5 MJ) and provides a more comprehensive evaluation through a four-step open-loop recycling route, substitution benefits against petroleum-based AC, assessment of energy sources, and comparison with commercial AC.

### 3.1. Structural and Chemical Characterization

To gain insight into the material’s structural nature and surface chemistry, X-ray diffraction (XRD) and Fourier-transform infrared spectroscopy (FTIR) analyses were conducted on the as-synthesized PCC obtained via both NaOH- and KOH-based activation routes.

**Figure 3:**
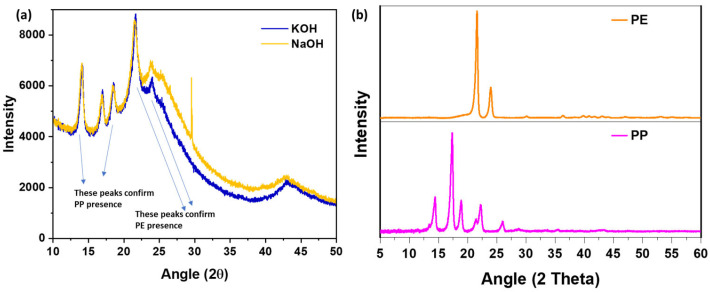
XRD patterns for (**a**) PCC using KOH and NaOH-activation routes, (**b**) pure PP and PE.

As shown in Figure 3, both PCC samples exhibit diffuse diffraction patterns lacking long-range crystalline order. Minor reflections around 13°, 17°, 19°, and 22° suggest residual PE and PP domains, while weak humps near 25° and 44° are characteristic of disordered carbon structures commonly observed in activated carbon. The XRD patterns of pure PP and PE exhibited sharp and intense peaks, reflecting their semi-crystalline structures. PP showed prominent reflections at 14.1°, 17.1°, 18.6°, and 21.3°, corresponding to the α-monoclinic crystalline phase, while PE displayed peaks at 21.5° and 23.9°, indicative of its orthorhombic crystalline structure. In contrast, the XRD spectra of the activated carbon flakes (PCCs) synthesized via KOH and NaOH activation routes revealed broad, low-intensity peaks centered around 23° and 43° (2θ), attributed to the (002) and (100) planes of amorphous carbon. The absence of sharp crystalline peaks confirmed the effective transformation of the polymers into amorphous carbon through chemical activation and carbonization.

**Figure 4:**
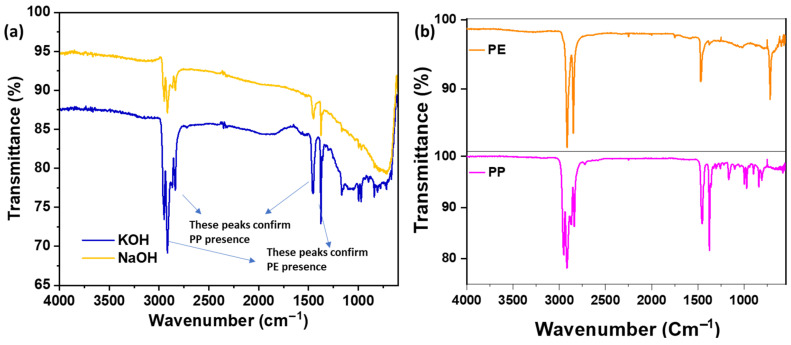
FTIR of (**a**) PCC using KOH and NaOH-activation routes showing polymer peaks, (**b**) pure PP and PE.

The FTIR spectra (Figure 4) reveal the presence of functional groups. Both KOH- and NaOH-activated samples exhibit characteristic absorption bands around 2850 cm^−1^ and 1400 cm^−1^, attributed to C–H stretching and bending vibrations from polyolefins, along with a distinct peak near 1100 cm^−1^ corresponding to AC [46]. Our previous studies have demonstrated these XRD and FTIR signatures for pure PE, pure PP, and their mixtures [47,48], which supports the identification of these domains in the current samples.

**Figure 5:**
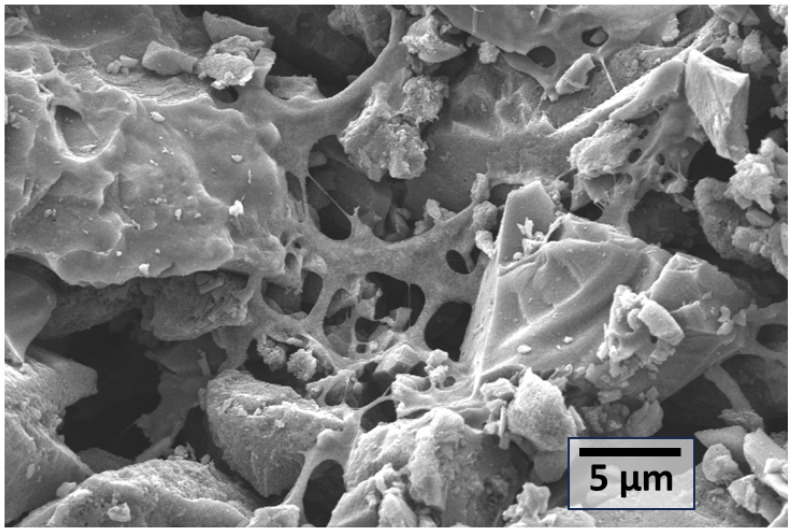
SEM image of polymer–carbon composite, reflecting polymer strands on carbon.

Figure 5 presents the SEM micrograph of the synthesized polymer–carbon composite (PCC), offering morphological evidence of the material’s heterogeneous structure. The image reveals an interconnected porous network with fragmented, rough carbonaceous surfaces interspersed with continuous, film-like regions and strand-like morphologies. These smooth, stretched domains are indicative of residual polymeric structures partially retained during chemical activation and carbonization. The observed microstructure supports the classification of the material as a polymer–carbon composite rather than a purely amorphous activated carbon. The SEM analysis thus complements the XRD findings and reinforces the conclusion that the final material comprises coexisting carbon and polymeric domains.

### 3.2. Contribution Analysis

**Figure 6:**
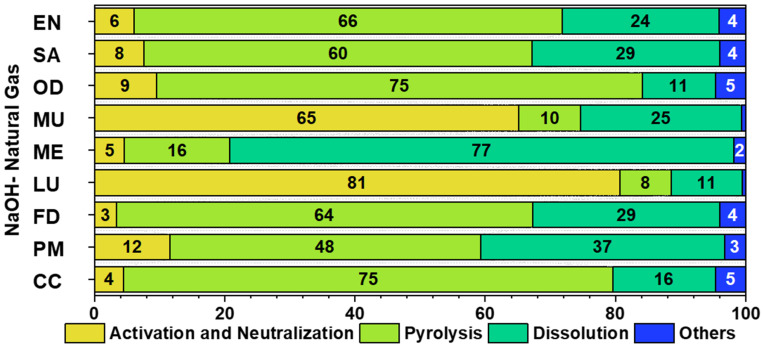
Contribution analysis of various steps involved in the production of PCC.

Figure 6 illustrates the contribution analysis, identifying the stages with the most significant EI where improvements can be made to produce PCC. Among these, Activation and Neutralization, Pyrolysis, and Dissolution are the key processes that heavily influence environmental indicators.

Pyrolysis emerges as the most critical stage in terms of EI, particularly concerning CC and EN, contributing 75% to CC and 66% to EN. This process consumes 18.14 MJ of energy to produce PCC. Additionally, pyrolysis leads to the release of various gases and by-products, which can contribute to environmental pollution [8,49,50]: Carbon Dioxide (CO_2_), Volatile Organic Compounds (VOCs), Carbon Monoxide (CO), Methane (CH_4_), and Particulate Matter (PM). The primary emissions from pyrolysis—CO_2_ and CH_4_—are significant contributors to CC due to their greenhouse gas properties. Strategies such as emission reduction or gas capture for energy recovery could help mitigate some of these EI. The Activation and Neutralization stage is the second most crucial step in PCC production, accounting for 6% of EN and 4% of CC. This process involves treating dried PF powder with an activation agent (KOH or NaOH in this study), followed by neutralization through water washing, which specifically requires 15 L of water. The Dissolution phase also plays a significant role in EI, contributing 24% to EN and 66% to CC. The energy-intensive nature of material dissolution significantly increases carbon emissions, primarily due to the use of xylene as a solvent and the high energy demand of vacuum technology. The solvent extraction and subsequent condensation process for recovery further elevate EI due to their substantial energy consumption. Finally, all other steps combined contribute 4% to EN and 5% to CC, making them relatively minor compared to the key stages mentioned above.

### 3.3. EI of Various Energy Sources to Produce PCC

**Figure 7:**
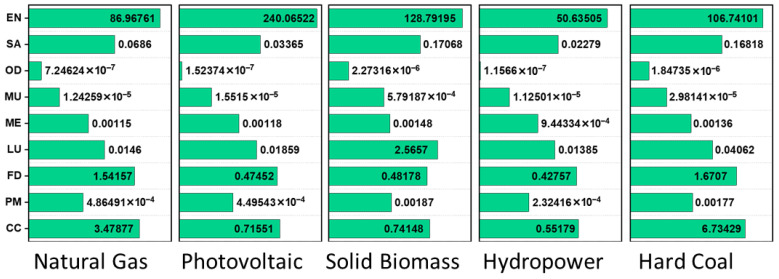
EI associated with the production of PCC (NaOH) using various energy sources.

The selection of an energy source significantly influences the EI of PCC production, particularly in terms of EN and CC impact. The results in Figure 7 indicate considerable variations in both metrics across different energy sources.

EN Analysis: Among the five energy sources analyzed, photovoltaic (PV) energy exhibits the highest energy demand (240 MJ), followed by solid biomass (128.79 MJ), hard coal (106.74 MJ), natural gas (86.97 MJ), and hydropower (50.63 MJ). The high EN values for PV and biomass suggest that these sources require more energy-intensive infrastructure and processing. Photovoltaic energy’s high EN can be attributed to the energy-intensive manufacturing of solar panels and associated systems, despite its low operational emissions. Solid biomass also shows a relatively high EN, likely due to the energy required for biomass collection, transportation, and combustion. Hard coal and natural gas have moderate energy demands, reflecting the energy required for extraction, transportation, and combustion. Hydropower has the lowest EN (50.63 MJ), making it the most energy-efficient option in this assessment.

CC Analysis: The CC impact, measured in kg CO_2_-equivalent emissions, follows a different trend than EN. Hard coal exhibits the highest CC impact (6.73 kg CO_2_-eq), whereas hydropower has the lowest (0.55 kg CO_2_-eq), followed closely by photovoltaic (0.72 kg CO_2_-eq) and solid biomass (0.74 kg CO_2_-eq). Hard coal’s high CC impact is expected due to the direct combustion of carbon-rich fuel, leading to substantial CO_2_ emissions. Natural gas has a moderate CC impact (3.48 kg CO_2_-eq), primarily due to methane leakage and CO_2_ emissions during combustion. Photovoltaic and solid biomass energy sources have relatively low CC impacts, despite their high EN values, because they do not rely on direct fossil fuel combustion. Hydropower emerges as the most environmentally favorable option, with both the lowest EN and CC impact. Its minimal emissions result from the absence of fuel combustion, although lifecycle impacts such as dam construction and land use changes should be considered.

Comparison and Sustainability Implications: The trade-off between low CC emissions and high EN consumption is evident in renewable sources such as photovoltaic and biomass. While these sources contribute less to climate change, they require significant energy input for infrastructure and fuel processing. Conversely, fossil fuels like coal and natural gas have moderate to high EN and much higher CC emissions, making them less sustainable options.

From a sustainability perspective, hydropower appears to be the most efficient and environmentally friendly option, exhibiting the lowest EN and CC impact. Photovoltaic energy, despite its high EN demand, remains a viable alternative due to its low CC emissions. However, when considering long-term sustainability, a combination of renewable sources with energy-efficient production methods could further reduce the environmental footprint of PCC production.

These results highlight the need for careful selection of energy sources in PCC production to minimize EI. While hydropower is the most favorable option, photovoltaic energy also offers a low-carbon alternative despite its higher energy demand. Reducing reliance on fossil fuels, particularly coal, would significantly lower the carbon footprint of PCC production. Future research could explore hybrid energy systems or energy recovery strategies to further optimize the environmental performance of PCC production processes.

### 3.4. Cost Considerations Based on Literature

The experiments were conducted in Qatar, where electricity is supplied from the national grid at a relatively low rate of approximately 0.0357 USD/kWh [51], leading to lower operational energy costs. Additionally, the Gulf region has an abundance of date palm trees, making palm fronds readily available. Combined with the ease of accessing plastic waste, the cost of raw materials is significantly reduced.

Previous studies [52,53] have established that producing AC from waste materials is economically favorable. Therefore, we believe the cost of AC flakes produced in this work would be lower and potentially competitive with commercial alternatives.

### 3.5. Comparison with Commercial AC

**Figure 8:**
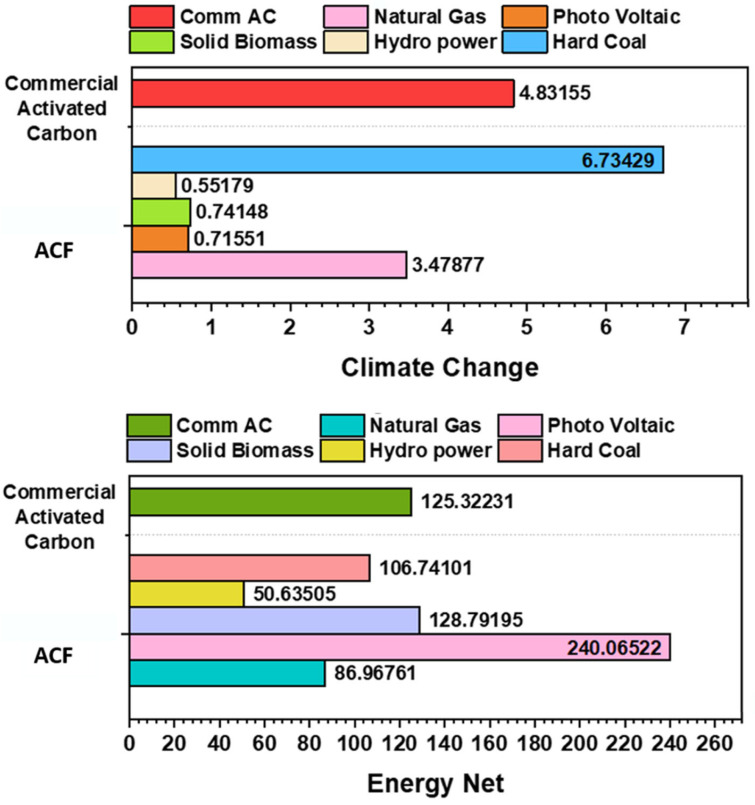
Comparison of PCC with commercial AC (climate change in kg CO_2_ eq. and energy net in MJ).

The LCA results highlight the significant environmental benefits of PCC production compared to commercial AC. Figure 8 presents a comparative analysis of PCC production across different energy routes alongside commercial AC. When using natural gas as a fuel, PCC production achieved a 28% reduction in climate change (CC) impact, lowering emissions from 4.83 kg CO_2_ eq. to 3.48 kg CO_2_ eq. per kg of AC. Additionally, net energy (EN) consumption decreased by 30%, from 125 MJ to 86.97 MJ per kg of AC, indicating improved energy efficiency. These findings demonstrate PCC’s potential as a more sustainable alternative to conventional AC, offering lower carbon emissions, reduced energy demand, and enhanced waste valorization.

### 3.6. Limitations and Uncertainties

This study focuses on the production of PCC while accounting for inherent limitations and uncertainties. (a) Laboratory data are used to generate the results, which may differ in commercial production. (b) Optimizing solvent efficiency and energy use during large-scale PCC production could reduce EI. (c) There may be a difference in recycling rates for water and xylene depending on changes in quality after reusing. (d) Power consumption may vary slightly due to differences in equipment specifications, such as those used in vacuum systems. (e) Greener solvents could replace the solvent used in this study, yielding lower EI. (f) Recycling of PCC is not addressed in this study. (g) The findings are specific to the Qatar region, as differences in energy mixes across countries can impact the results.

## 4. Conclusions

This study demonstrates the environmental sustainability of producing activated carbon flakes (PCC) from palm fronds (PF) and polyolefin waste through a simple four-step open-loop recycling process. By integrating biomass and plastic waste, the proposed method not only addresses the practical limitations of powdered activated carbon in aqueous systems but also promotes circular economy practices by valorizing two abundant waste streams.

The Life Cycle Assessment (LCA) results confirm that PCC production offers notable environmental advantages over conventional petroleum-based activated carbon. Specifically, it achieves a 28% reduction in climate change impact and a 30% decrease in net energy demand, with total values of 3.57 kg CO_2_ eq. and 88.59 MJ per kg of PCC, respectively. Pyrolysis emerged as the most energy- and emission-intensive stage. Among activation agents, NaOH was found to be more sustainable than KOH. Furthermore, substituting natural gas with renewable energy sources—particularly hydropower—substantially reduces both energy use and emissions, with hydropower yielding the lowest impacts (50.63 MJ and 0.55 kg CO_2_ eq.). Overall, this work provides a scalable and environmentally conscious approach for producing functional carbon materials while contributing to sustainable waste management and resource recovery.

## Figures and Tables

**Table 1:** Details of various steps involved in producing 1 kg of PCC.

Process	Resource Allocation		Remarks
	NaOH activation	KOH activation	
Transportation	2 km		Collection and transportation by diesel truck
Shredding	59.76 kJ		-
Drying	479.52 kJ		Drying under hot-air oven
Activation	136.36 g NaOH	190.9 g KOH	2M NaOH and 2M KOH
	1.704 L water	1.704 L water	
Neutralization	15.0 L water	15.0 L water	Washing with water using glass rod and checking pH till neutralized.
Drying	432 kJ		Drying under hot-air oven
Pyrolysis	18,144 kJ		
Dissolution	1296 kJ; 333.3 mL Xylene; 20 g UHMWPE + 10 g PE-waste + 10 g PP-waste		Dissolution was carried out on Heidolph hotplate magnetic stirrer for 60 min, at 130 °C.
Heat Treatment	135 kJ		Drying in hot-air oven

**Table 2:** Selected EI categories.

SN	Impact Category	Unit	Abbreviation
1.	Energy Net	MJ	EN
2.	Smog in Air	kg O_3_ eq.	SA
3.	Stratospheric Ozone Depletion	kg NOx eq.	OD
4.	Marine Eutrophication	kg N eq.	MU
5.	Marine Ecotoxicity	kg 1,4-DB eq.	ME
6.	Land Use	Annual crop eq.·y	LU
7.	Fossil Depletion	kg oil eq.	FD
8.	Particulate Matter	kg PM2.5 eq.	PM
9.	Climate Change	kg CO_2_ eq.	CC

**Table 3:** EI comparison of KOH vs. NaOH.

	EN	CC
NaOH	86.97	3.48
KOH	88.59	3.57

## Data Availability

All data generated or analyzed during this study are included in this published article.
